# The psychosocial aid response after the 22/03/2016 attacks in Belgium: a community case study

**DOI:** 10.3389/fpubh.2024.1362021

**Published:** 2024-03-08

**Authors:** Emilie Muysewinkel, Lara Vesentini, Helena Van Deynse, Lise Eilin Stene, Johan Bilsen, Roel Van Overmeire

**Affiliations:** ^1^Mental Health and Wellbeing Research Group, Vrije Universiteit Brussel, Brussel, Belgium; ^2^Public Health Department, Vrije Universiteit Brussel, Brussel, Belgium; ^3^Norwegian Centre for Violence and Traumatic Stress Studies, Oslo, Norway

**Keywords:** registration data, posttraumatic stress disorder, mental health, terrorism, mental healthcare

## Abstract

**Introduction:**

After the terrorist attacks, early psychosocial care is provided to people considered at risk of developing mental health issues due to the attacks. Despite the clear importance of such early intervention, there is very few data on how this is registered, who is targeted, and whether target-recipients accept such aid.

**Methods:**

Using registry data from the Centre General Wellbeingwork (CAW), a collection of centers in the regions Brussels and Flanders that provide psychosocial care, we examined the early psychosocial care response after the terrorist attacks of 22/03/2016 in Belgium.

**Results:**

In total, 327 people were listed to be contacted by the CAW, while only 205 were reached out to (62.7%). Most were contacted within a month (84.9%), and were victims of the attacks (69.8%). Overall, the majority was female (55.6%).

**Conclusion:**

Overall, target recipients were witnesses and survivors of the attacks, though a large proportion of people were not reached by the early outreach.

## Introduction

On March 22, 2016, terrorists carried out bombings at the national airport and a metro station in Belgium. These attacks resulted 35 people dead and left hundreds more wounded (1). Such terrorist attacks can lead to long-term psychological problems among those directly exposed (2, 3). Among such mental health issues are post-traumatic stress disorder (PTSD), depression and anxiety disorders (4–6). Furthermore, such disorders can be associated with other problems, such as drug abuse, alcohol abuse, social relationship problems, suicidal ideation… (7–10). In general, only a minority of individuals will develop mental health issues as a result of their exposure to terrorist attacks (2, 11, 12). For example, 6 months after the 1995 Oklahoma City Bombing in the United States, around 33% of those directly exposed had PTSD. Seven years later this was 26%, while 18 and a half years later, this was still around 23.2% (13–15). So, while the majority tends to recover, for this still large minority professional mental health assistance is required, and if possible, as soon as possible (2, 16).

Governments often organize psychosocial outreach efforts following such disasters (17, 18). For example, after the terrorist attacks in Norway, outreach was extended to those identified as survivors of the attacks, their families and the bereaved (19). However, there is currently no available information regarding the target recipients of outreach efforts following the terrorist attacks in Belgium, including who accepted assistance and received follow-up support.

Such information is crucial for enhancing psychosocial care plans for future disasters. Presently, it seems that the establishment of these plans are poorly based on evidence, international guidelines or a framework (17). Despite the availability of international guidelines, a comparative study of Norway, France, and Belgium revealed substantial differences in the plans and outreach strategies following terrorist attacks (18). Still, there is currently little evidence-based knowledge on the best practices for providing early psychosocial care after disasters (17). However, understanding how psychosocial outreach is currently implemented is vital for shaping future appropriate psychosocial care planning. Therefore, this study seeks to provide insights into the psychosocial outreach efforts following the terrorist attacks in Belgium.

Therefore, we aim:

1. To describe the early psychosocial outreach after a terrorist attack.
2. To describe how many people receive this early outreach.
3. To provide information on their background.

## Methods

In this registry data study, we studied data from the Centrum Algemeen Welzijnswerk (CAW) (roughly translated as “Centre General Wellbeingwork”). This is a collection of centers in the regions Brussels and Flanders that provide psychosocial care, but also financial aid, legal aid, *et cetera*. The CAW also organizes psychosocial outreach after events such as disasters (called “*Slachtofferhulp*” in Dutch, meaning “Victim Aid”). In theory, this care should be provided within 3 days after a potentially traumatic event.

For the terrorist attacks on March 2016, the role of the CAW was described as being the first line aid for victims of the attacks, screening of post-traumatic stress disorder (PTSD), identifying of the needs of victims, and helping victims transferring to further professional aid if necessary (20, 21). It was thus, for the regions of Flanders and Brussels, one of the most important organizations in terms of providing psychosocial outreach and care (20). For the psychosocial outreach, first, CAW received information on who to contact from the police services, through so-called dispatch lists (20). Then, CAW-Victim Aid contacted the persons on this list, providing them a choice of further follow-up at the CAW or other services. Of course, persons could also refuse any further aid from the CAW.

### Data

**Figure 1:**
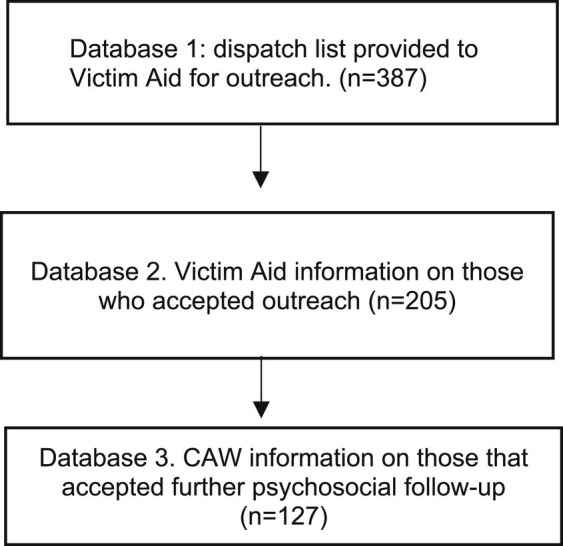
Steps of including cases in study.

For analysis, we used three different CAW databases (see Figure 1), which encompass data from all centers in Belgium, regarding the individuals affected by the attacks in March 2016.

In the first database, we examined a list of individuals intended to be contacted and those who were actually contacted. This list contained solely information on how many should be contacted per region, and how many were contacted.

The second database contains information on all persons that have been contacted by the CAW-Victim Aid in 2016 (i.e., accepted the outreach). It uses a standardized system where the reason for contact is labeled. Because the terrorist attacks were labeled as ‘disaster’ we filtered on this term, adding the specific date of March 22, 2016 to select our cases for this study. For these cases, we were able to identify demographics, such as gender, age, the place where the CAW met the person, country of origin, and whether they accepted further follow-up through the CAW or referral to other services. In the case of referral to other services, there was no information on what services these were. The role of the person in the attacks was registered in an open text field. The role indicated in what capacity the person was exposed. For example, they could be family members of someone wounded at an attack, a witness… Our selection was validated by ensuring it matched the number of cases listed by the CAW (refer to step 1). ID numbers for each included case (made by the CAW as unique identifiers) were checked so that no doubles were included.

In the third database, information is available on those who accepted further follow-up of the CAW. Identification occurred through cross-referencing with the previous database, using unique identifiers avoid the inclusion of duplicates. Demographics included the persons’ age, gender, and place of residence (Brussels, outside Brussels, or unknown). The reason for their follow-up by the CAW was also recorded through standardized categories. We filtered on those related to mental health: “mental health/wellbeing,” “coping issues,” and “issues related to traumatic events.”

In all variables, “unknown” indicated missing information.

### Analysis

We used descriptive statistics to look at demographics of who was contacted. Microsoft Excel and SPSS 27.0 were employed.

### Ethics statement

This study was approved by the Ethics Committee of the UZ Brussels/VUB (B.U.N. 143201836345).

## Results

### Outreach

**Figure 2:**
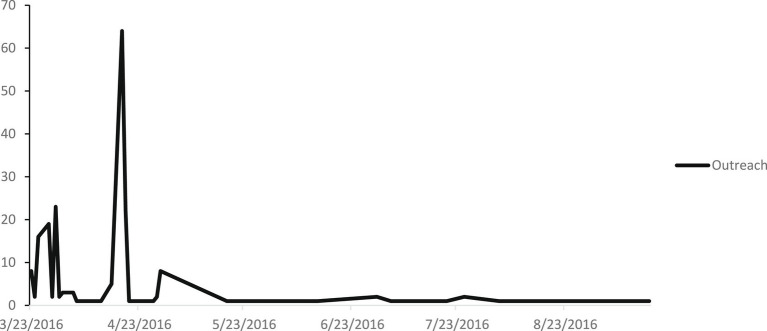
Outreach.

In total, 327 people were listed to be contacted by the CAW, while only 205 were actually reached out to (62.7%). Among those who were contacted, most were contacted within a month after the attacks (84.9%) (see Figure 2). Those contacted included victims, relatives of victims (such as partners and parents) and witnesses. Most of them were female, between 26 and 59 years old, and were labeled as victims of the attacks (see Table 1).

**Table 1:** Outreach.

	*N* = 205	%
**Gender**		
Male	77	37.6
Female	114	55.6
Unknown	14	6.8
**Age**		
12–17	11	5.4
18–25	28	13.7
26–59	128	62.4
60+	10	4.9
Unknown	28	13.7
**Country of origin**		
Belgium	49	23.9
Other EU-country	3	1.5
Non-EU country	11	5.4
Unknown	142	69.3
**Role in attacks**		
Victim	143	69.8
Relative	15	7.3
Witness	4	2.0
Other	10	4.8
Unknown	33	16.1
**Referral**		
Further to CAW	127	62.0
Refused offer	18	8.8
Other service	55	26.8
Unknown	5	2.4

### Follow-up by CAW

**Figure 3:**
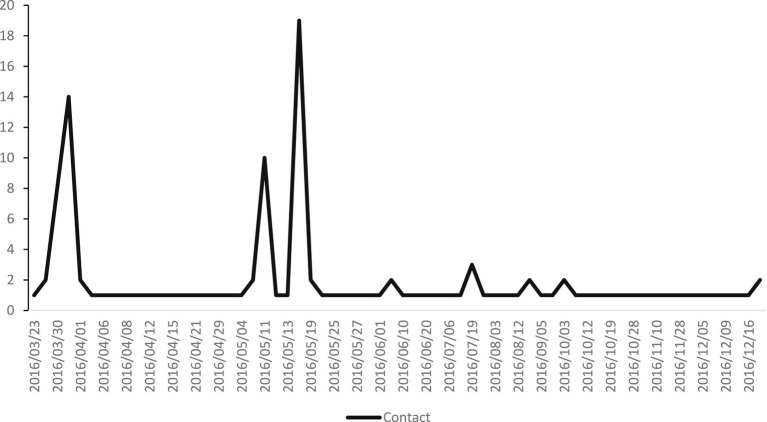
Further follow up at CAW.

Of these 205 people who were contacted by the CAW-Victim Aid, 127 were referred to the centra of the CAW. Eighteen did not accept the offer of referral and 55 were referred to another service. Most who accepted further aid from the CAW were female (66.9%) (see Table 2). All 127 had contact with the CAW for reasons related to mental wellbeing, coping issues, and issues related to a traumatic experience. By the end of May, 63.8% of the 127 had received follow-up at the CAW-Victim Aid (see Figure 3).

**Table 2:** Further psychosocial aid at CAW.

	*N* = 127	%
**Gender**		
Male	39	30.7
Female	85	66.9
Unknown	3	2.4
**Age**		
15–17	1	0.8
18–25	24	18.9
26–59	89	70.1
60–79	8	6.3
Unknown	5	3.9
**Home of victim**		
Brussels	93	73.2
Outside Brussels	15	11.8
Unknown	19	15.0

## Discussion

This study showed unique data on the psychosocial outreach after the attacks of March 2016 in Belgium. First, 327 people were on dispatch lists to be contacted. However, only 62.7% had contact with someone of the CAW. Contact with the majority of this group was established within a month. Those contacted were mostly female and between 26 and 59 years old. Of those that were contacted, 8.8% refused further aid from the CAW. Of those that accepted further CAW-aid, most lived in Brussels, were between 26 and 59 years old and female and all had issues with coping, their mental health and wellbeing and issues related to a traumatic experience.

While 327 people who were supposed to be contacted might seem low (considering that a metro and airport were attacked at rush hour), it should be noted that personnel such as rescue workers (e.g., police officers, soldiers, firefighters, *et cetera*) in theory received mental health aid from inside their organization (18). These will not have been included in the dispatch list. Additionally, this outreach was not aimed at the French-speaking population, who had a separate outreach. Furthermore, the role of the CAW seems to have been defined restricted to “directly exposed” people (20). This can explain why so few relatives were contacted (7.3%). However, relatives of victims, whether they were deceased or not, can also develop severe mental health issues, might have need for psychosocial care, or even legal advice. For example, Norwegian studies show that bereaved parents as well as parents of survivors equally need psychosocial care (22, 23). In that sense, it should be questioned whether the psychosocial outreach should be broader.

The current study shows that even with contact information, it is quite difficult to have full outreach. A proportion of people that were supposed to be contacted did not seem to have any contact with the CAW-Victim Aid. This may have several reasons. First, it can be due to the way of registration. It is possible that these people were initially approached but simply refused contact with the CAW and that only those that experienced issues accepted the outreach attempt. Another issue might be the generally low trust in government associated institutions in Belgium, which might have led to refusal of accepting aid (24).

A second reason is that the CAW might have simply been overloaded and unable to contact everyone. In fact, qualitative research does seem to indicate that this might be correct, where some victims indeed received aid from the CAW and others not (21). A report from a governmental audition also seemed to indicate that the planning of psychosocial outreach was uneven and insufficient (18). This indicates that the CAW was unable to contact everyone that they were supposed to contact. This would also explain why the psychosocial outreach occurred over a period of a month, and not, as is usual for the CAW, in the period of 3 days. However, those that were reached and wished further follow-up, received this follow-up fast. By the end of May, 2 months after the attacks, the majority of people had received follow-up from the CAW. Therefore, the aid provided to those that accepted further follow-up, was swift.

Another important finding is, ironically, the lack of findings, as the data in the databases were often incomplete, which resulted in a lot of missing data. In terms of improving the preparedness of a country for future disasters and terrorist attacks, more accurate and comprehensive data is essential. For example, there was no registered information on how a person was contacted (e.g., in person, online, telephone). It might be expected that employees of the CAW will have this information for their specific clients. Yet, in terms of improving the utility of such data on early psychosocial outreach, such information should be registered. Other points of improvement are, first, the registry of mental health aspects. Despite the use of a PTSD-questionnaire, there is no such registry data on mental health aspects. Yet, through such data, a better estimation of the severity of the issue could be made. In addition, there is, to our knowledge, no study on the PTSD-prevalence among the survivors of the Belgium-attacks (21).

Secondly, information on the referrals would be useful, with more information on why aid was refused, or the reasons why certain survivors could not be reached. This is important for psychosocial outreach planning. After all, it might be due to practical issues. For example, the addresses and names may potentially not have been properly noted on the dispatch list, which would indicate that this needs to be improved. Another explanation could be that the CAW staff did not manage to properly earn the trust of survivors, perhaps due to a lack of experience. For example, in qualitative research, it was noted that the inexperience of employees of the CAW often discouraged survivors to accept aid (21). Considering the poor state of psychotraumatology in Belgium, this hypothesis does not seem unreasonable (20).

Third, a major question on disaster outreach, is the timing. It is quite normal for in the days after an attack to feel severe stress. A large-scale population study in the week after the attacks showed that depressive and anxiety symptoms were relatively high. However, such emotions generally fade. For example, after sexual assault, 90% of survivors show PTSD-symptoms. After 3 months, this percentage is 40–50% (25). On the other hand, bureaucratic issues, such as trying to get reimbursements from insurance funds, start almost immediately. With limited resources, the ethical question is: what takes priority, aiding with legal issues, or aiding with mental health issues? Because, the CAW of course has limited resources, and cannot provide aid for one person within a week, and then again within a month, when PTSD can be diagnosed according to the DSM-5 (26).

It seems that the CAW mainly takes the decision to wait almost a month for first contacts. Indeed, it might be defended from a mental health perspective. After all, most people will have natural resilience (11, 27), and do not need professional mental health. However, considering the issues that survivors had with legal aspects of the terrorist attack, the question remains whether it is the right approach. Yet, this was also very specific for the terrorist attacks, as terrorist attacks are unexpected, while fires in homes, robberies or even sexual assault are part of what the CAW usually handles, but also what insurance funds are used to.

Considering the difficulties that survivors had to maneuver the legal aspects of the attacks, to prove they were indeed injured mentally, and to get back their personal belongings if they were left at the scene, it might be stated that helping with legal aspects early on is a form of mental health aid (21). Therefore, it seems most important for organizations such as the CAW to aid in all legal issues of such survivors first and provide all information possible so that survivors can reach out on their own to professional mental health aid. If possible, outreach would of course be repeated, but the situation after the attacks has shown that this is quite difficult—in fact, it is difficult even to reach everyone a first time.

However, such questions also show the ethical decisions that need to be made. Knowing that very early response within the first days might not be useful with regards to mental health, the question might be whether it is necessary. An argument against this, is that such early response shows that policy makers are concerned with the issues that people have experienced. Social acknowledgement has been shown to be an important aspect of the development of PTSD (28, 29). Either way, this is a choice policy makers have to make and should be recognized as part of disaster medicine, where with limited resources the most “economic” choices have to be made.

A fourth point that remains unclear after viewing the data, is with regards to logistics and practicalities. As in: how were survivors contacted? This could be done through telephone, in-person, or perhaps through other ways. The datasets do not provide an answer on that. However, considering the workings of the CAW, it was probably a combination of telephoning and in-person visits. Furthermore, qualitative research on the survivors seems to confirm this (21). However, a lot will depend on the information police services noted. If the information is unclear, finding the survivors might be more difficult. For example, if telephone numbers are not registered, contacting the survivors might be more difficult.

Registering the method of contacting would also inform policy on the best way to reach out to survivors noted on dispatch lists. For example, perhaps in the days after the attacks, survivors do not want to be contacted by phone numbers they do not recognize, or do not want to let people from the CAW in their home. Furthermore, it is important to look at the hours that contact was attempted. While seemingly a small aspect, people who work nightshifts would be difficult to contact during day-hours, for example. Such information would greatly inform the pro-active outreach of the CAW after disasters.

The information provided in this study is unique in several ways. First, it provides data on who was considered some kind of victim, together with demographics, which was missing in international research on the Belgian attacks (21). Second, it provides invaluable information for the field of psychosocial disaster planning, as the few studies that exist on psychosocial outreach generally view the outreach from the side of the survivor, and not on the registration of survivors. There are several aspects noted in this study that are important to take into account for future disasters. After all, preparation is everything (30), but to be prepared, countries also need to make sure that they can learn from the experiences of disasters and improve upon their pro-active outreach.

This study is limited in the sense that many people probably received psychosocial care through other means, or simply did not want aid (21). Furthermore, we could not include the French speaking of Belgium, as they have a different organization of psychosocial outreach. Finally, more detailed information was unavailable from the CAW representatives, preventing us from filling up gaps where necessary. Of course, as quite some time has passed now, there would be difficulties recreating the exact actions of the CAW. This again emphasizes the point that proper registration is necessary, so that the outreach can be evaluated and be improved for future disasters. After all, disasters do not occur regularly. Therefore, registering as much as possible, in case of a disaster, is of the upmost importance.

Further studies should continue looking at registry data of the outreach after disasters. It provides invaluable information about the organization of aid, potential delays due to a lack of personnel and/or resources, or the impossibility of reaching everyone that organizations want to reach. Naturally, survey studies are also necessary to judge the quality of the aid.

## Data availability statement

The data analyzed in this study is subject to the following licenses/restrictions: this is registry data from the CAW. To access data, approval of the CAW will be needed. Requests to access these datasets should be directed to roel.van.overmeire@vub.be.

## Ethics statement

The studies involving humans were approved by Ethics Committee of the UZ Brussels/VUB. The studies were conducted in accordance with the local legislation and institutional requirements. Written informed consent for participation was not required from the participants or the participants’ legal guardians/next of kin.

## Author contributions

EM: Conceptualization, Data curation, Formal analysis, Funding acquisition, Investigation, Methodology, Project administration, Resources, Software, Validation, Visualization, Writing – original draft, Writing – review & editing. LV: Conceptualization, Formal analysis, Investigation, Methodology, Resources, Supervision, Validation, Writing – review & editing. HD: Conceptualization, Formal analysis, Investigation, Methodology, Supervision, Writing – review & editing. LS: Formal analysis, Methodology, Project administration, Software, Supervision, Writing – review & editing. JB: Formal analysis, Methodology, Project administration, Resources, Software, Writing – review & editing. RVO: Conceptualization, Data curation, Formal analysis, Project administration, Supervision, Writing – original draft, Writing – review & editing.
